# Elevated glucose metabolism via the hexosamine biosynthesis pathway: A metabolic signature of high-fluorodeoxyglucose-uptake lung adenocarcinoma

**DOI:** 10.1007/s00595-026-03276-2

**Published:** 2026-03-10

**Authors:** Hikaru Watanabe, Hideki Makinoshima, Naoki Kanauchi, Takanobu Kabasawa, Jun Suzuki, Satoshi Takamori, Takayuki Sasage, Kohei Abe, Kazumasa Hoshijima, Tetsuro Uchida, Tomoyoshi Soga, Satoshi Shiono

**Affiliations:** 1 Department of Surgery II, Faculty of Medicine, Yamagata University, 2-2-2 Iida-Nishi, Yamagata, 990-9585 Japan; 2 Department of General Thoracic Surgery, Nihonkai General Hospital, Sakata, Japan; 3 Tsuruoka Metabolomics Laboratory, National Cancer Center, Tsuruoka, Tsuruoka Japan; 4 Shonai Regional Industry Promotion Center, Tsuruoka, Japan; 5 Division of Translational Information, Exploratory Oncology Research & Clinical Trial Center, National Cancer Center, Kashiwa, Japan; 6 Department of Pathological Diagnostics, Faculty of Medicine, Yamagata University, Yamagata, Japan; 7 Institute for Advanced Biosciences, Keio University, Tsuruoka, Japan; 8 Human Biology-Microbiome-Quantum Research Center, Keio University, Tsuruoka, Japan

**Keywords:** Metabolome analysis, Hexosamine biosynthesis pathway, Warburg effect, Lung adenocarcinoma, 18-fluoro-2-deoxyglucose positron emission tomography

## Abstract

**Purpose:**

To evaluate hexosamine biosynthetic pathway (HBP) involvement in high-fluorodeoxyglucose (FDG)-uptake lung adenocarcinoma.

**Methods:**

We conducted metabolomic analysis to evaluate the HBP in patients with lung adenocarcinoma, who underwent preoperative 18-FDG positron emission tomography. Capillary electrophoresis-time-of-flight mass spectrometry was done to obtain 511 small-molecule metabolite spectra, and a principal component analysis was performed.

**Results:**

We examined 80 tissue samples: 40 tumor-adjacent non-tumor tissue samples and 40 resected lung adenocarcinomas. The principal component analysis confirmed good clustering between the tumor and non-tumor tissues. The non-tumor tissues comprised uniform materials, whereas the tumor tissues comprised a mixture of materials. Heatmaps for 50 metabolites revealed lower glucose and citrate levels and higher levels of lactate, glycolysis metabolites, succinate, fumarate, adenosine di- and -monophosphate, and all essential amino acids in the tumor tissues than the non-tumor tissues. HBP intermediate and uridine diphosphate N-acetylglucosamine levels were also higher in the tumor tissues. Both lactate and HBP intermediate levels were higher in hypermetabolic tumor tissues (standardized uptake value ≥ 3) than in non-hypermetabolic tumor tissues (standardized uptake value < 3). Low-FDG-uptake cells showed strong expression for glucose transporter SLC2A1 and weak expression for O-linked N-acetylglucosamine, whereas high-FDG-uptake cells showed strong expression for both markers.

**Conclusions:**

Hypermetabolic adenocarcinoma may be associated with intensified glycolysis and HBP activation.

## Introduction

Non-small cell lung cancer (NSCLC) remains prevalent and continues to have a high mortality rate [1]. Recently, advances in sensitive radiologically diagnostic techniques, such as high-resolution computed tomography (CT), have enabled the early detection of NSCLC when surgical treatment is feasible [2]. However, recurrence develops in approximately 10% of patients with surgically resected early-stage NSCLC [3]. Because the characteristics of NSCLC are not heterogeneous, further advances in the treatment of early-stage NSCLC are needed.

18 F-deoxyglucose positron emission tomography (FDG-PET)/CT plays an important role in identifying highly aggressive NSCLC. Quantitative evaluation using the maximum standard uptake value (SUVmax) in PET/CT reflects glucose metabolism within tumors and the malignancy of lung cancer [4, 5]. Several studies have demonstrated that SUVmax for PET/CT is a surrogate to identify tumor aggressiveness and is useful for predicting the pathological invasiveness of early-stage lung adenocarcinoma [6–8] and other cancers [9, 10]. Hypermetabolic NSCLC, which shows higher SUVmax on PET/CT, tends to have pathologically invasive factors and a worse prognosis.

Malignant tumors are characterized by a metabolism altered from that of benign tissues [11–13]. The unique metabolic features of cancer cells support abnormal survival and growth characteristics by increasing the energy available to the cells and macromolecular precursors and reducing equivalents [14–16]. Metabolome analysis is a promising method to enhance our understanding of metabolic regulation in cancer cells and identifying biomarkers for early-stage cancers [12, 17, 18]. Warburg observed that cancer cells convert glucose to lactate in the presence of an adequate oxygen supply, a phenomenon known as the Warburg effect or aerobic glycolysis [19]. Defects in mitochondrial respiration may underlie the aerobic glycolysis in cancer cells [20]. Indeed, the Warburg effect is the basis for tumor imaging via FDG PET/CT [21]. Upregulated aerobic glycolysis is considered a hallmark of cancer [22].

Regarding the metabolic pathways in cancer cells, the hexosamine biosynthetic pathway (HBP) uses glycolytic pathway intermediates as substrates and plays a role in the development of the malignant properties of cancer cells [23]. However, the role of this pathway in lung adenocarcinoma and its association with high FDG uptake remain unclear. Considering the biological behavior and metabolism of lung cancer, this study aimed to elucidate how HBP is involved in hypermetabolic lung adenocarcinoma by performing a metabolomic analysis and exploring new therapeutic targets for high-grade lung adenocarcinoma.

## Materials and methods

### Ethical statements

All experiments were performed in accordance with the study protocol approved by the Nihonkai General Hospital Institutional Review Committee (Institutional Review Board (IRB) No. 28-5-2; 30 January, 2017) and the Yamagata University Faculty of Medicine (IRB No. 2022 − 331; 7 April, 2023). Informed consent was obtained from all participants. This study was performed in accordance with the principles of the Declaration of Helsinki.

### Patients’ backgrounds and specimen collection

Non-tumor and tumor tissues obtained from patients with lung adenocarcinoma, who underwent surgery between February, 2017 and January, 2018, were used as surgical samples. During this 1-year period, consent was obtained from 40 patients. We recorded the following patient characteristics: age, sex, smoking history, stage, and mutation status (Table 1). Non-tumor tissues were taken from the part of the excised specimen farthest from the tumor. To ensure the stability of the metabolomic profile, tissue samples were collected as soon as possible. The time from resection of the pulmonary vessels (the onset of warm ischemia) to the removal of the specimen from the body was within approximately 60 min. After removal, tumor and non-tumor tissues were dissected immediately on a chilled tray and snap-frozen. All frozen samples were then transferred promptly to a deep freezer (− 80 °C) for long-term storage.

**Table 1:** Baseline clinicopathological characteristics of the patients enrolled in the study

**Number of patients**	40
**Age**,** median [IQR]**,** years**	70 [64–77]
**Sex**,** n (%)**	
Male	21 (52.5%)
Female	19 (47.5%)
**Smoking status**,** n (%)**	
Non-smoker	24 (60.0%)
Smoker	16 (40.0%)
**SUVmax**,** median [IQR]**	2.60 [1.46–7.13]
**Histology**,** n (%)**	
Adenocarcinoma	40 (100%)
**International Association for the Study of Lung Cancer Grade**,** n (%)**	
1	19 (47.5%)
2	18 (45.0%)
3	3 (7.5%)
**Lymphovascular invasion**,** n (%)**	
Present	13 (32.5%)
**Visceral pleural invasion**,** n (%)**	
Present	10 (25.0%)
**Pathological stage**,** n (%)**	
0	4 (10%)
I	28 (70%)
II	3 (7.5%)
III	4 (10.0%)
IV	1 (2.5%)
**Mutation status**,** n (%)**	
*EGFR* mutation	18 (45%)
*ALK* mutation	1 (2.5%)

IQR, interquartile range; SUVmax, maximum standardized uptake value; EGFR, epidermal

growth factor receptor; ALK, anaplastic lymphoma kinase

**Fig. 1:**
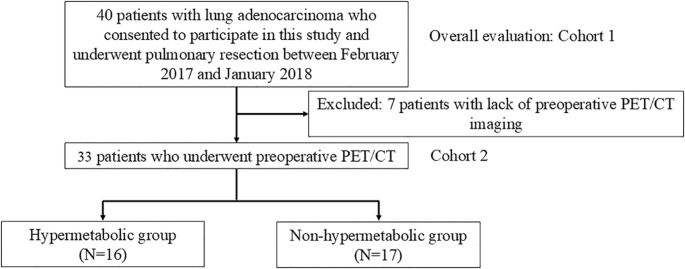
Patient flowchart PET/CT, positron emission tomography/computed tomography

This study compared the metabolic profiles of patients who underwent surgery and PET/CT. According to a previous study, patients with radiologically aggressive lung cancers (defined as tumors with SUVmax ≥ 3.0) had a significantly worse prognosis than those with radiologically non-aggressive tumors (SUVmax < 3.0) [24]. While various thresholds have been proposed, depending on the facility, we adopted this value as a clinically relevant cutoff for identifying invasive potential in our NSCLC cohort [24]. To evaluate whether HBP activation depends on this specific threshold, we analyzed the correlation between SUVmax as a continuous variable and key HBP metabolites. Radiologists established a region of interest for measuring SUVmax, which was then calculated automatically using software. Figure 1 presents a flowchart summarizing patient inclusion in the study. We excluded seven patients who did not undergo preoperative PET/CT.

The TNM Classification of Malignant Tumors (8th edition) was used for tumor staging [25], and lung adenocarcinomas was classified histologically in accordance with the International Association for the Study of Lung Cancer/American Thoracic Society/European Respiratory Society classification [26].

### Metabolite quantification in tissues using capillary electrophoresis-time-of-flight mass spectrometry (CE-TOFMS)

For metabolite extraction, pre-weighed deep-frozen samples (~ 50 mg each) were homogenized by a cell disrupter (Shake Master Neo, Bio-Medical Science Co., Ltd., Tokyo, Japan) after the addition of 500 µL of methanol that contained internal standards (20 µmol/L each of L-methionine sulfone, 2-(N-morpholino)-ethanesulfonic acid, and D-camphor-10-sulfonic acid). The homogenate was then mixed with 200 µL Milli-Q water (Sigma-Aldrich, Poole, UK) and 500 µL chloroform and centrifuged at 4600 g for 15 min at 4 °C. Subsequently, the aqueous solution was filtered centrifugally through a 5-kDa cutoff filter (Human Metabolome Technologies, Tsuruoka, Japan) for protein removal. The filtrate was concentrated centrifugally and dissolved in 50 µL of Milli-Q water containing the reference compounds (200 µmol/L each of 3-aminopyrrolidine and trimesate) immediately before CE-TOFMS (Agilent Technologies, Palo Alto, CA, USA) analysis. Intracellular metabolites were measured by CE-TOFMS, as described previously [27, 28].

### Data analysis and statistical analysis

The raw data were processed using our proprietary software (MasterHands, Tsuruoka, Japan) [28]. For each sample, the measured metabolite concentrations were normalized using the tissue weight to obtain the amount of metabolite contained per gram of each sample. The results are presented as the mean ± standard deviation, unless indicated otherwise. Statistical analysis and principal component analysis were performed and a heatmap diagram was created using MetaboAnalyst (https://www.metaboanalyst.ca/) [29–31]. The p-values obtained from the Student’s t-test were corrected using the Benjamini–Hochberg method [30]. A corrected p-value threshold was calculated based on a false discovery rate of 5%. Only metabolites with a corrected p-value (q-value) < 0.05 were considered significantly different. Metabolite set enrichment analysis (MSEA) was performed in accordance with published protocols [32–34]. Briefly, MSEA was performed using the metabolic profiles of surgically resected tumor samples. Metabolic profiles of surgically resected hypermetabolic tumor samples (SUVmax ≥ 3.0) and non-hypermetabolic tumors (SUVmax < 3.0) were classified biochemically. Metabolites exhibiting more than a two-fold variation and *p* < 0.05 in Welch’s t-test were used for MSEA. The Kyoto Encyclopedia of Genes and Genomes was used to classify metabolites and metabolic pathways.

### Pathological examination of lung adenocarcinoma tissues

Immunohistochemical staining was performed to investigate, from a histopathological perspective, the relationship between the HBP and glucose metabolism in cancer cells within hypermetabolic tumors (SUVmax ≥ 3.0). We examined the protein expression of glucose transporter SLC2A1 (aka GLUT1) and O-linked N-acetylglucosamine (O-GlcNac) by immunohistochemistry using a lung adenocarcinoma tissue microarray (*n* = 6). Three samples each of hypermetabolic tumors with SUVmax ≥ 3.0 and non-hypermetabolic tumors with SUVmax < 3.0 were selected. With IRB approval of the Yamagata University Faculty of Medicine in accordance with US Common Rule, patient samples were retrieved from the surgical pathology service at the Department of Pathology, Yamagata University Faculty of Medicine. Immunohistochemical staining was performed with BOND RXm (Leica Biosystems, Nussloch, Germany) in accordance with the manufacturer’s protocol. We used primary antibodies that were specific for Glut1 (E461; rabbit IgG; Cell Signaling Technology, Boston, MA, USA) and O-GlcNAc (RL2; mouse IgG1; FUJIFILM Wako Pure Chemical Corporation, Osaka, Japan).

## Results

Table 1 summarizes the patients’ characteristics. We compared 80 tissue samples, 40 of which were surgically derived from tumor-adjacent non-tumor tissues and 40 of which were surgically resected lung adenocarcinomas. Surgical samples allow for comparison between matched tumor and non-tumor pairs of tissues. Thus, to confirm whether metabolome analysis could be performed properly, first we analyzed snap-frozen samples of surgically resected non-tumor (*n* = 40) and tumor (*n* = 40) tissues. Small-molecule metabolite spectra were obtained using CE-TOFMS, and pattern‑recognition multivariable statistical analysis was used to screen for different metabolites. A total of 239 and 272 metabolites (total = 511) were assessed using negative- and positive-ion mass spectrometry, respectively. Of these, 107 metabolites exhibited significant differences between non-tumor and tumor samples (*p* < 0.05 and q < 0.05) (Supplementary Table S1).

**Fig. 2:**
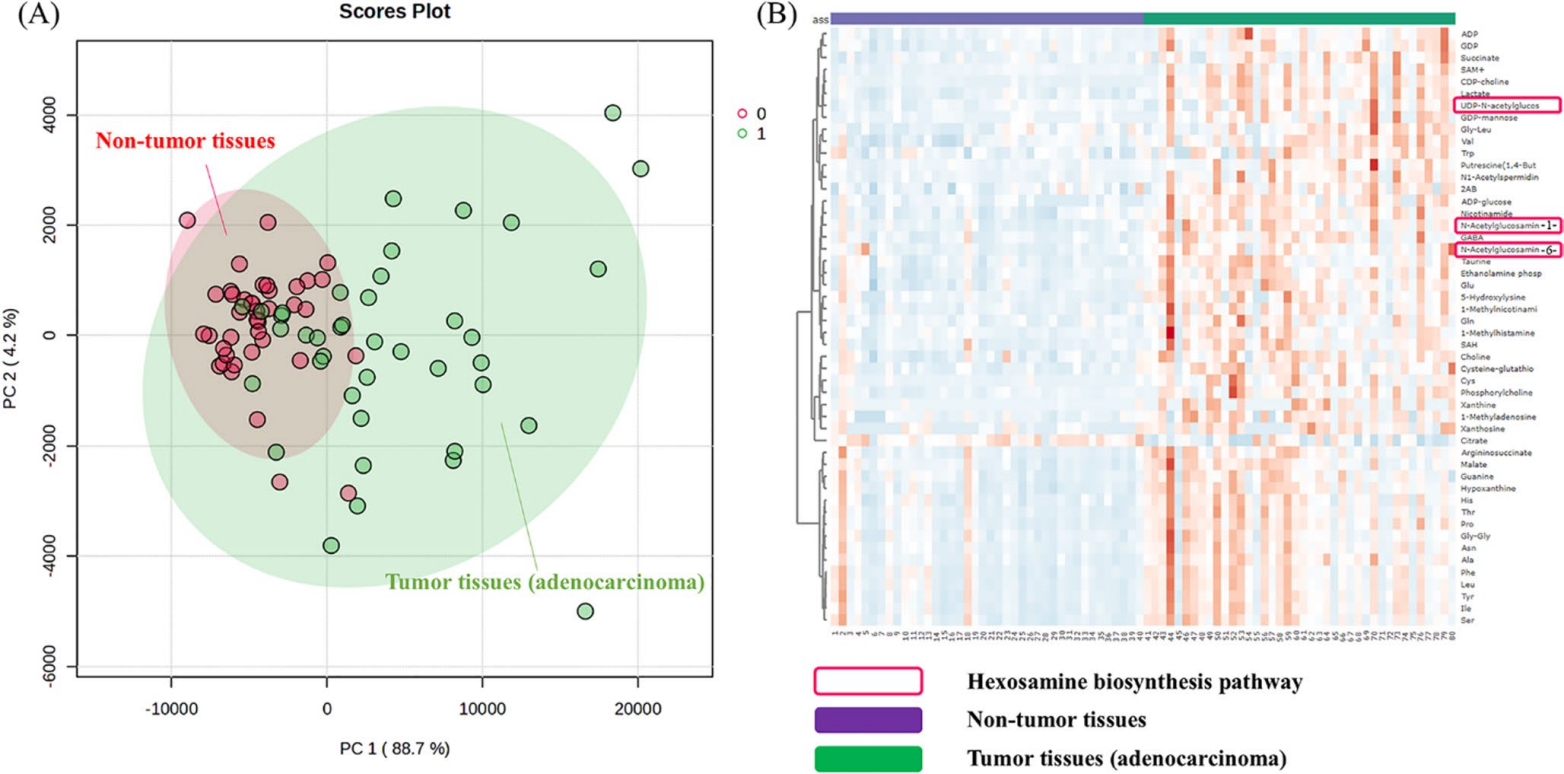
**a**. Principal component analysis score plots showing all identified metabolites in tumor-adjacent non-tumor tissues and 40 surgically resected lung adenocarcinomas. **b**. A clustered heatmap showing metabolic profile differences between tumor-adjacent non-tumor tissues and lung adenocarcinoma tissues. Tumor-adjacent non-tumor tissues are shown in purple (*n* = 40), and lung adenocarcinoma tumor tissues are shown in green (*n* = 40). Metabolites are shown in rows. Individual values are coded by color, ranging from blue (minimum) to red (maximum). Metabolites that reached significance (*p* < 0.05 after Benjamini-Hochberg correction) are highlighted in the text and marked with asterisks (*) in Supplementary Table S1. In the heatmap, all 50 representative metabolites are displayed to provide biochemical context, with significant metabolites contributing to the distinct clustering of tumor versus non-tumor tissues. PCA, principal component analysis; ADP, adenosine diphosphate; GDP, guanine diphosphate; SAM+, S-adenosyl methionine-positive; CDP-choline, cytidine-5-diphosphocholine-choline; UDP-N-acetyl glucose, uridine diphosphate-N-acetylglucosamine; Gly-Leu, glycine-leucine; Val, valine; Trp, tryptophan; 1,4 diaminobut, 1,4 diaminobutane; GABA, gamma-aminobutyric acid; Glu, glucose; 1-methylnicotinami, 1-methyl nicotinamide; N1-acetylspermidin, N1-acetylspermidine; 2AB, 2aminobenzamide; N1-acetylglucosamin-1, N-acetylglucosamine-1; ethanolamine phosph, ethanolamine phosphate; N-acetylglycosamin-D, N-acetyl-α-d-glycosamine; Gln, glutamine; SAH, s-adenosylhomocysteine; cystine-glutathio, cystine-glutathione; Cys, cystine; His, histidine; Thr, threonine; Pro, proline; Gly, glycine; Asn, asparagine; Ala, alanine; Phe, phenylalanine; Tyr, tyrosine; Ile, isoleucine; Ser, serine

We performed principal component analysis on the peaks extracted from all experimental samples. The principal component analysis score plots showed that the pooled tumor-adjacent non-tumor tissues and the 40 surgically resected lung adenocarcinomas clustered well. The non-tumor tissues comprised uniform samples, whereas the tumor tissues comprised a mixture of materials (Fig. 2a). To identify significant metabolic differences while examining broader metabolic patterns, a heatmap was generated based on hierarchical clustering analysis using 50 metabolites (Fig. 2b). Glucose levels were lower in the tumor tissues of lung adenocarcinoma than in adjacent tissues. However, the levels of glycolysis intermediates, such as glucose 6-phosphate, fructose 1,6-bisphosphate, and glycerol 3-phosphate, were higher in the lung adenocarcinoma tumor tissues than in adjacent non-tumor lung tissues (Fig. 2b). Increased glucose availability was accompanied by an increase in upstream glycolytic intermediates. The level of the end-product of glycolysis, lactate, was higher in adenocarcinoma tissues than that in non-tumor lung tissues (Fig. 2b). Citrate levels were lower in lung non-tumor tissues than in lung tumor tissues; however, succinate and fumarate levels were significantly higher in lung adenocarcinoma tissues than in adjacent tissues (Fig. 2b). Regarding adenylate energy charge, adenosine di- and -monophosphate and all essential amino acid levels were higher in lung adenocarcinoma tissue than in non-tumor tissue. Additionally, the levels of HBP intermediates, such as N-acetylglucosamine-1-phosphate and N-acetylglucosamine-6-phosphate, as well as the HBP end product, uridine diphosphate (UDP)-N-acetylglucosamine, were higher in lung adenocarcinoma tissue than in non-tumor tissue (Fig. 2b). Supplementary Table S1 shows all 107 metabolites that exhibited different levels in surgical-tumor versus non-tumor tissues. These results are consistent with those of previous studies, suggesting that the surgical specimen samples in this study were managed under optimal storage conditions.

The accumulation of FDG, as measured by SUVmax on PET/CT, was compared among lung adenocarcinoma patients according to their clinical characteristics, histologic subtypes, and pathological stages (Table 2). Table 2 shows that the hypermetabolic group (SUVmax 3.0) consisted predominantly of high-grade (G3) tumors, while the non-hypermetabolic group was characterized by low-grade (G1) tumors. More specifically, the G3 components in the hypermetabolic tumors were identified primarily as solid and micropapillary subtypes. In contrast, the G1 tumors in the non-hypermetabolic group were predominantly of the lepidic subtype. This histological distribution demonstrates that the activation of the HBP signature is closely linked to these clinically aggressive histological patterns.

**Table 2:** Baseline clinicopathological characteristics of the patients who underwent preoperative positron-emission tomography and computed tomography

	Hypermetabolic group	Non-hypermetabolic group	*p*-value
**Number of participants, n**	16	17	
**Age**,** median [IQR]**,** years**	71 [62–78]	70 [63–76]	0.527
**Sex**,** n (%)**			0.166
Male	11 (68.8%)	7 (41.2%)	
Female	5 (31.2%)	10 (58.8%)	
**Smoking status**,** n (%)**			0.084
Non-smoker	6 (62.5%)	12 (70.6%)	
Smoker	10 (37.5%)	5 (29.4%)	
**SUVmax**,** median [IQR]**	7.13 [5.38–8.20]	1.58 [1.10–1.88]	<0.001
**Histology**,** n (%)**			
Adenocarcinoma	16 (100%)	17 (100%)	
**IASLC Grade**,** n (%)**			<0.001
1	1 (6.3%)	12 (70.6%)	
2	12 (75.0%)	5 (29.4%)	
3	3 (18.7%)	0 (0%)	
**Lymphovascular invasion**,** n (%)**			0.004
Present	10 (62.5%)	2 (11.8%)	
Absent	6 (37.5%)	15 (88.2%)	
**Visceral pleural invasion**,** n (%)**			<0.001
Present	9 (56.3%)	0 (0%)	
Absent	7 (43.7%)	17 (100%)	
**Pathological stage**,** n (%)**			<0.001
0	0 (0%)	1 (5.9%)	
I	9 (56.2%)	15 (88.2%)	
II	2 (12.5%)	1 (5.9%)	
III	4 (25.0%)	0 (0%)	
IV	1 (6.3%)	0 (0%)	
**Mutation status**,** n (%)**			
EGFR mutation	2 (12.5%)	13 (76.5%)	
ALK mutation	0 (0%)	1 (5.9%)	

PET, positron-emission tomography; CT, computed tomography; IQR, interquartile range; SUVmax,

maximum standardized uptake value; EGFR, epidermal growth factor receptor; ALK, anaplastic lymphoma kinase

**Fig. 3:**
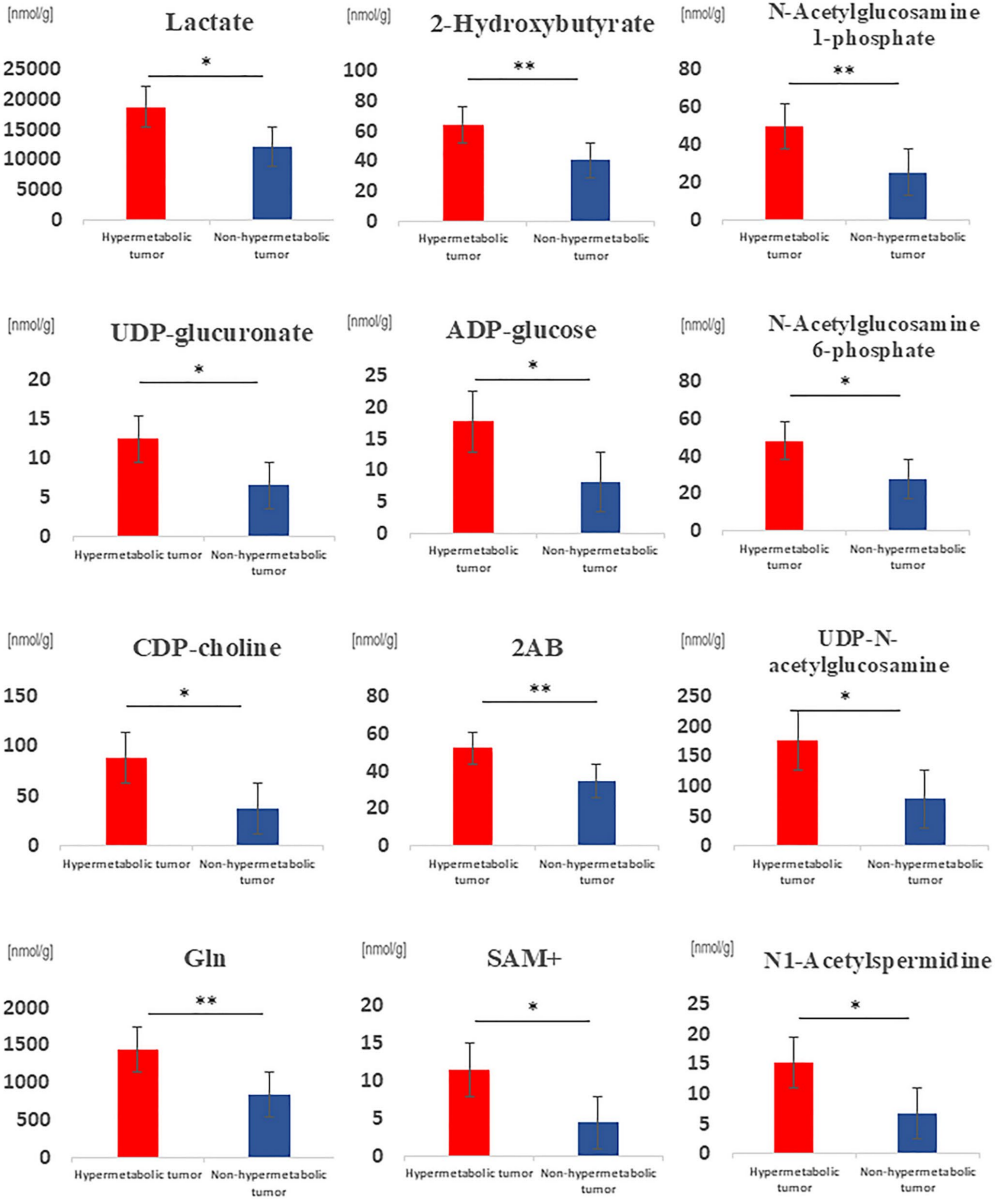
Different metabolic profiles between hypermetabolic lung adenocarcinoma and non-hypermetabolic lung adenocarcinoma tissues. The average amounts (nmol/g) of hypermetabolic tumor tissue (red bar, *n* = 16) versus non-hypermetabolic tumor tissue (blue bar, *n* = 17) are shown. Error bars indicate the mean ± standard deviation. *, *p* < 0.05; **, *p* < 0.001 versus control by two-tailed Student’s t-test UDP, uridine diphosphate; ADP, adenosine diphosphate; CDP, cytidine-5-diphosphocholine; 2AB, 2aminobenzamide; Gln, glutamine; SAM+, S-adenosyl methionine-positive

Figure 3 shows representative metabolites exhibiting different FDG uptake levels in surgically resected hypermetabolic tumors (SUVmax ≥ 3.0) and non-hypermetabolic tumors (SUVmax < 3.0). The Y-axis indicates the metabolite concentration per gram of sample (nmol/g). The levels of lactate, which is the end-product of upregulated aerobic glycolysis, were 1.5-fold higher in hypermetabolic tumor tissues than in non-hypermetabolic tumor tissues. Levels of HBP intermediates, such as N-acetylglucosamine-1-phosphate and N-acetylglucosamine-6-phosphate, as well as the HBP end-product, UDP-N-acetylglucosamine, were higher in hypermetabolic tumor tissues than in non-hypermetabolic tumor tissues (Fig. 3). To examine the relationship between FDG-uptake intensity and HBP flux without relying solely on a fixed threshold, we performed a Spearman’s correlation analysis. We found that SUVmax, when treated as a continuous variable, correlated positively with several key HBP metabolites, including UDP-N-acetylglucosamine (*r* = 0.47, *p* < 0.01), N-acetylglucosamine-1-phosphate (*r* = 0.57, *p* < 0.01) and N-acetylglucosamine-6-phosphate (*r* = 0.51, *p* < 0.01) (Supplementary Fig. S1). This indicates that HBP activation is progressively intensified in proportion to the degree of FDG accumulation.

**Fig. 4:**
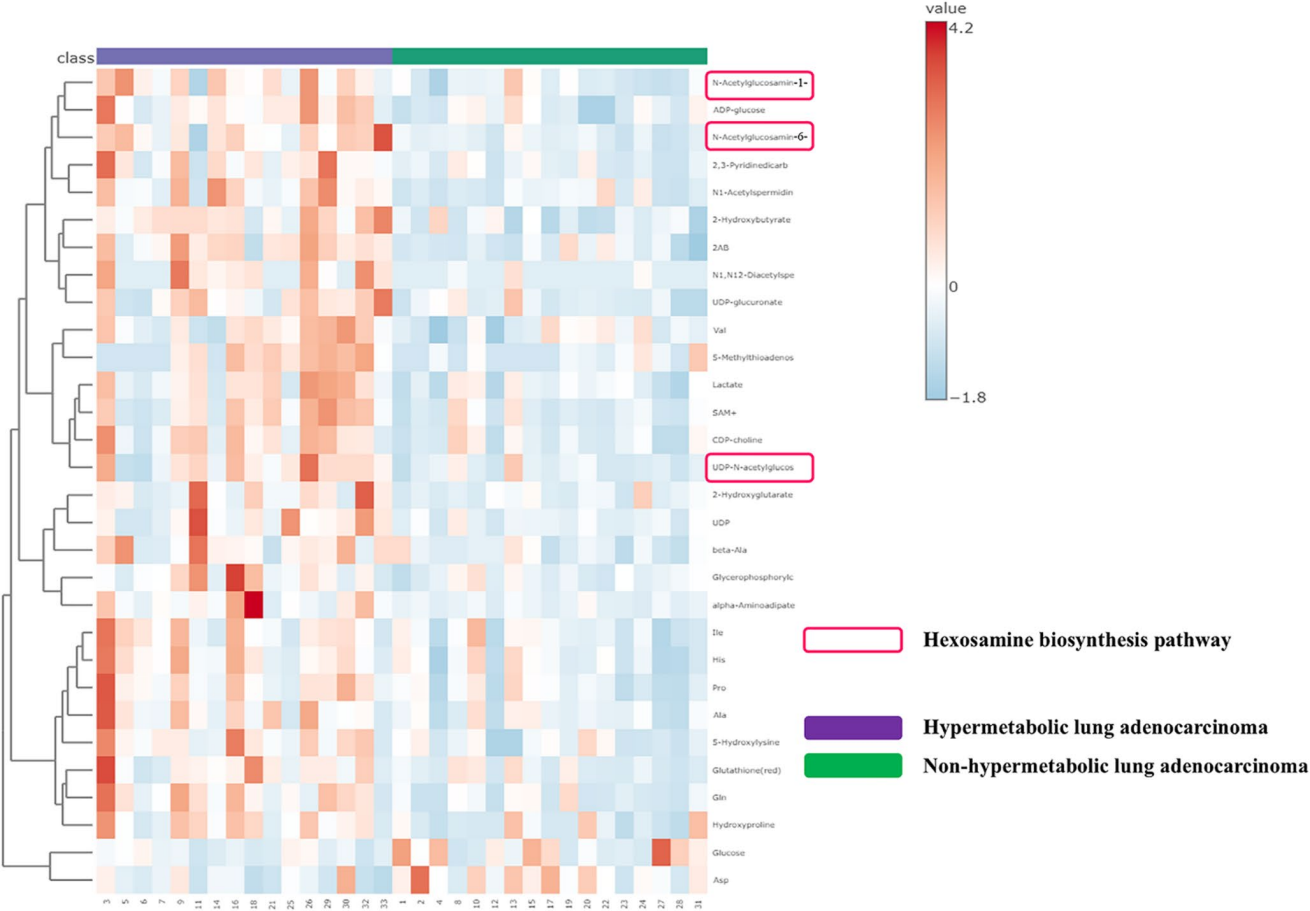
Clustered heatmap showing the metabolic profile differences between hypermetabolic and non-hypermetabolic lung adenocarcinoma tissues. The hypermetabolic lung adenocarcinoma tissues are shown in purple (*n* = 16) and the non-hypermetabolic lung adenocarcinoma tissues are shown in green (*n* = 17). Metabolites are shown in rows. Individual values are coded by color, ranging from blue (minimum) to red (maximum). This heatmap displays 12 significant metabolites (*p* < 0.05) with 18 contextual metabolites to illustrate the broader metabolic patterns. Significant metabolites are discussed primarily in the Results section to ensure transparency. N-acetylglycosamin-D, N-acetyl-α-d-glycosamine; ADP, adenosine diphosphate; N-acetylglycosamin-6, N-acetyl-glucosamine-6; 2,3-Pyridinedicarb, 2,3-pyridinedicarboxylate; N1-Aceytylspermidin; N1-acetylspermidine; 2AB, 2aminobenzamide; N1,N2-Diacetylspe, N1,N2-Diacetylspermine; Val, valine; 5-Methylthioadenos, 5-methylthioadenosine; SAM+, S-adenosyl methionine-positive; CDP-choline, cytidine-5-diphosphocholine-choline; UDP, uridine diphosphate; beta-Ala, beta-alanine; Glycerophosphoryl, glycerophosphorylcholine; Ile, isoleucine; His, histamine; Pro, proline; Ala, alanine; Gln, glutamine; Asp, asparagine

To identify significant metabolic differences while examining broader metabolic patterns, a heatmap was generated based on the hierarchical clustering analysis using 30 metabolites. The metabolites comprised the 12 significant, differentially expressed metabolites, as well as an additional 18 non-significantly different metabolites to provide context. Clustering was performed using Ward’s linkage method in hierarchical clustering. In Fig. 4, both metabolites (rows) and samples (columns) were clustered independently to visualize distinct metabolic patterns and clear sample separation.

**Fig. 5:**
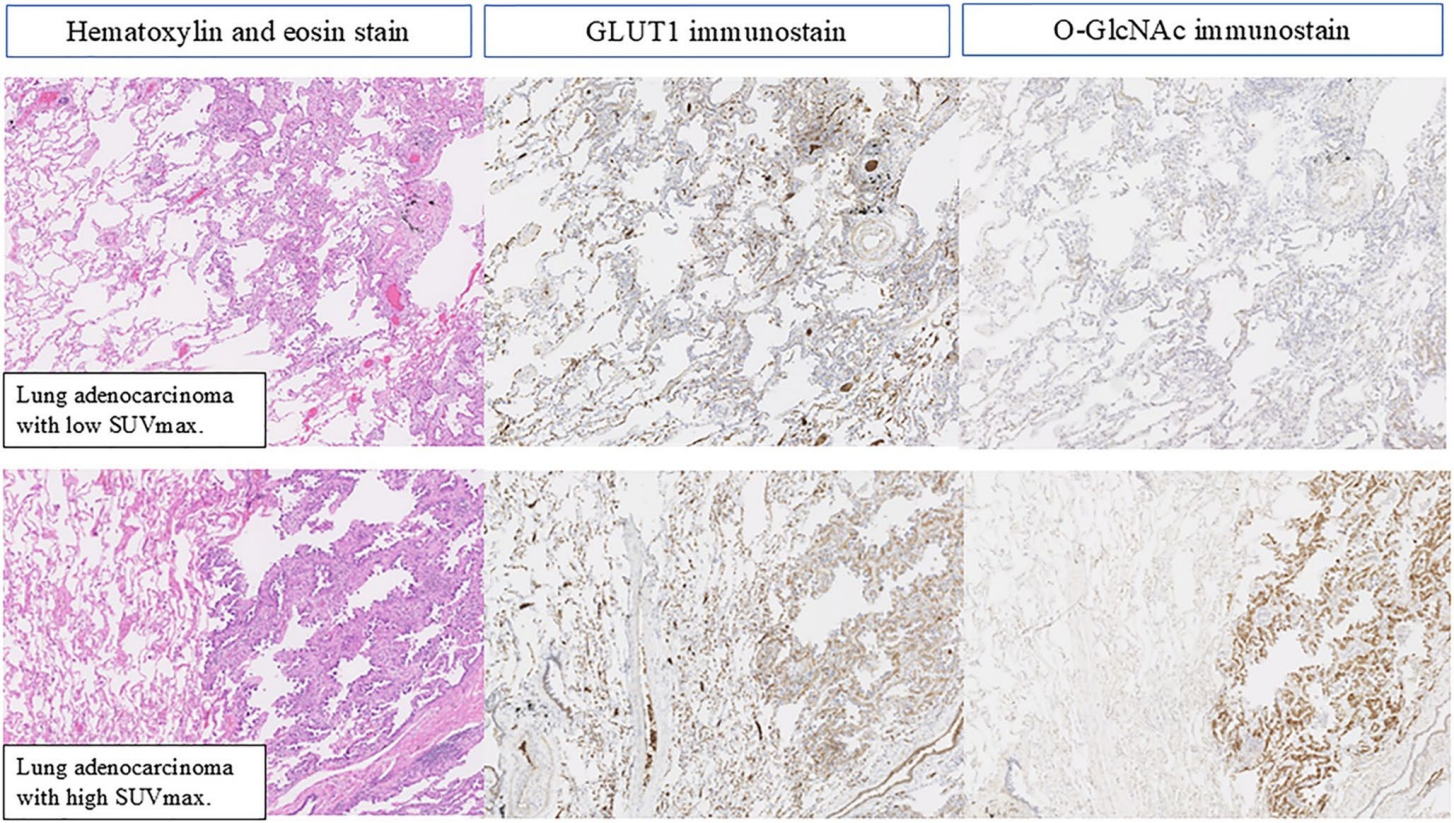
Representative samples of GLUT1 and O-GlcNAc expression. A comparison of lung adenocarcinoma with low SUVmax and that with high SUVmax. The left half of each histological image shows the non-tumor area and the right half shows the lung adenocarcinoma tumor area. O-GlcNAc expression is visible in cancer cells with a high SUVmax GLUT1, glucose transporter 1; O-GlcNAc, O-linked N-acetylglucosamine; SUVmax, maximum standardized uptake value

The HBP produces UDP-N-acetylglucosamine (UDP-GlcNAc), which is the substrate for O-GlcNAc modification, a key post-translational modification in cellular signaling. Immunohistochemical staining revealed that cells with low FDG uptake showed strong GLUT1 expression and weak O-GlcNAc expression, whereas cells with high FDG uptake showed strong expression for both GLUT1 and O-GlcNAc (Fig. 5).

## Discussion

We performed a comprehensive metabolomic analysis of surgically resected lung adenocarcinoma tissue to elucidate the metabolic basis of tumors with high FDG uptake. The key finding of this study is that hypermetabolic lung adenocarcinoma is characterized not only by the expected enhancement of aerobic glycolysis (the Warburg effect), but also by significant concomitant activation of the HBP, resulting in increased O-GlcNAc modification. This finding establishes elevated HBP flux as a distinct metabolic signature of aggressive lung adenocarcinoma with high FDG uptake.

A previous meta-analysis established that overall survival and disease-free survival were significantly shorter for NSCLC patients with high versus low SUVmax [35]. While several studies found a significant decrease in disease-free survival in NSCLC patients with a high SUVmax, there was no difference in patients with squamous cell carcinoma (SCC) [36, 37]. Currently, the results for FDG accumulation in lung cancer histologic subtypes are equivocal; some studies showed high FDG accumulation specifically in SCCs and others showed no difference in FDG accumulation among various lung cancer histologic subtypes [38–40]. A possible explanation for such diverse study results is that FDG accumulation depends on the GLUT1 transporter. GLUT1 is mainly located on the membrane of SCCs, which facilitates FDG uptake, unlike when GLUT1 is in the cytoplasm of tumor cells, as can be the case in adenocarcinomas [41].

**Fig. 6:**
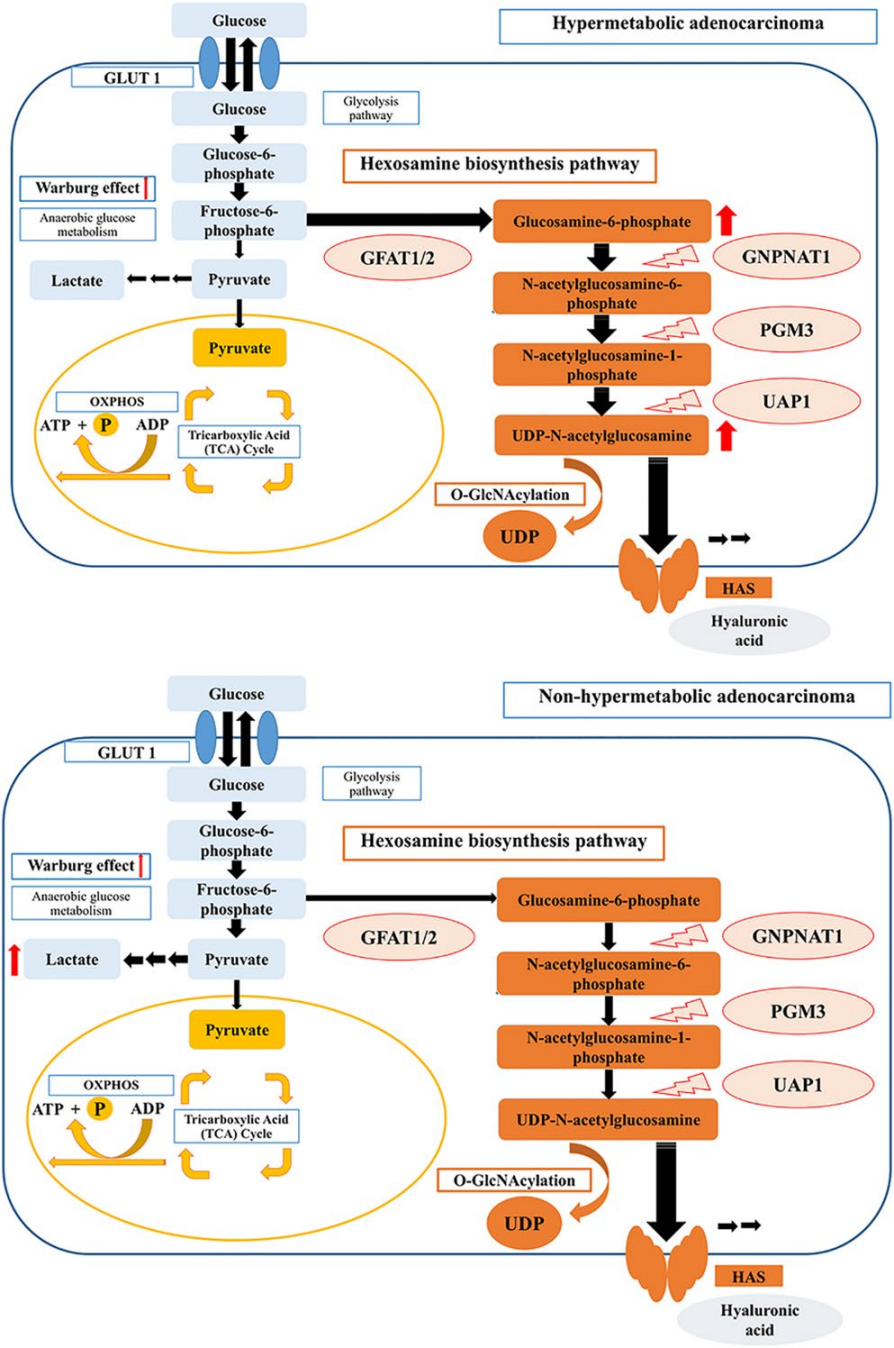
Schematic illustration of intracellular glucose metabolism and the hexosamine biosynthetic pathway GLUT1, glucose transporter SLC2A1; OXPHOS, Oxidative Phosphorylation; GFAT 1/2, glutamine fructose-6-phosphate amidotransferase 1/2; GNPNAT1, glucosamine-phosphate N-acetyltransferase 1; PGM3, phosphoglucomutase 3; UAP1, uridine phosphate-N-acetylglucosamine pyrophosphorylase; O-GlcNac, O-linked N-acetylglucosamine; UDP, uridine diphosphate; HAS, hyaluronan synthase; ATP, adenosine triphosphate; ADP, adenosine diphosphate; P, phosphorous

HBP is a critical intracellular glucose metabolic pathway involved in the synthesis of nucleotide-activated derivatives of glucose and protein glycosylation [42]. Typically, only a small percentage (3%–5%) of the glucose taken up by cells enters this pathway (Fig. 6). Key enzymes in the HBP, such as glutamine fructose-6-phosphate amidotransferase 1 (GFAT1), catalyze the conversion of fructose-6-phosphate and glutamine to glucosamine-6-phosphate, which is then converted to UDP-GlcNAc [43]. UDP-GlcNAc produced by the HBP is used for glycosaminoglycan synthesis, N-linked glycosylation, and O-GlcNAcylation. O- and N-glycosylation are tightly regulated post-translational modifications that influence protein structure, stability, and function [44]. In cancer, the dysregulation of O-glycosylation has been associated with various aspects of tumor progression and metastasis [45].

Our CE-TOFMS data showed a significant increase in key HBP intermediates and the end-product, UDP-GlcNAc, particularly in tumors with a high SUVmax (Fig. 3), which confirms that this pathway is dramatically upregulated in aggressive lung adenocarcinoma and suggests that activating the HBP is necessary to increase FDG uptake in lung adenocarcinoma. These findings align with those of previous studies demonstrating the importance of HBP in lung cancer aggressiveness [42, 43]; however, to our knowledge, this study is the first to use metabolite quantification in tissues via CE-TOFMS analysis of lung adenocarcinoma.

The involvement of the HBP in the development and progression of lung cancer has been studied extensively, providing insights into the molecular mechanisms underlying the role of the HBP in lung cancer pathogenesis [46]. In the initial rate-limiting step of the HBP, the conversion of fructose-6-phosphate to glucosamine-6-phosphate is catalyzed by GFPT1/2 enzymes. Recently, Wei et al. reported a correlation between the expression of GFAT1 [43] or GFAT2 [47], the rate-limiting enzyme of the HBP, and a poor prognosis for patients with lung adenocarcinoma. The final product of the HBP pathway, UDP-GlcNAc, inhibits GFAT via feedback inhibition [48]. When O-GlcNAcylation is activated, large quantities of the substrate UDP-GlcNAc are consumed, necessitating an acceleration of metabolic flux in glycolysis and HBP. This metabolic reprogramming was demonstrated functionally by the immunohistochemical results in our study. Low-FDG-uptake tumors showed strong GLUT1 expression but weak O-GlcNAc expression, whereas high-FDG-uptake tumors showed strong expression for both GLUT1 and O-GlcNAc (Fig. 5). This suggests that the high glucose uptake by GLUT1 does not merely end in lactate production but is actively channeled into the HBP for O-GlcNAc synthesis. The necessity of this HBP activation is supported further by the feedback regulation mechanism: high UDP-GlcNAc consumption for O-GlcNAcylation relieves feedback inhibition of the rate-limiting enzyme, GFAT, thereby sustaining high flux through both glycolysis and the HBP (Fig. 6). This finding corroborates recent evidence linking GFAT1 or GFAT2 expression to poor prognosis for lung adenocarcinoma [43, 47]. Furthermore, using clinical specimens obtained directly from surgery represents a significant strength for drug discovery. Unlike cell lines or animal models, our metabolomic data reflect the actual metabolic environment of human lung adenocarcinoma. Identifying HBP activation in these real-world samples provides a more reliable basis for developing HBP-targeted therapies, such as GFAT inhibitors, which ensures that these potential treatments address the metabolic reality of human tumors.

The limitations of this study include its small sample size (*n* = 40) and potential selection bias, as all samples were collected from a single center. The small number of cases also limited our ability to perform comprehensive multivariable or survival analyses, which could impact the generalizability of our proposed “metabolic signature”. However, we were able to perform metabolome analysis on surgically obtained specimens that were managed under highly optimized conditions. When determining the most suitable method for assessing levels of lung cancer metabolites, one should consider the advantages and disadvantages of surgically resected specimens. An advantage of using surgical specimens is that non-tumor tissue can be compared with tumor tissue, whereas a disadvantage is the time required to collect the specimen after stopping blood flow. Based on the metabolome analysis of tumor and non-tumor tissue samples in this study, which showed consistent metabolic clustering (Fig. 2a), we conclude that the samples were maintained under appropriate conditions. To address the limitations of this study, future studies should focus on several critical areas to translate the implications of the HBP signature into clinical applications. Specifically, the HBP signature must be validated in large independent cohorts to definitively confirm its prognostic value and clinical utility through rigorous multivariable modeling. Moreover, it is essential to investigate precisely which specific oncogenic or tumor-suppressive proteins are stabilized or functionally altered by the increased O-GlcNAc modification observed in lung adenocarcinoma with high FDG uptake. Such rigorous mechanistic validation is essential to establish GFAT or O-GlcNAc transferase as a viable therapeutic target that can disarm this aggressive metabolic phenotype and pave the way for targeted therapeutics.

In conclusion, this study suggests that hypermetabolic lung adenocarcinoma may be accompanied not only by enhanced glycolysis via the Warburg effect, but also by marked activation of the HBP and a subsequent increase in O-GlcNAc modification. This finding is a critical factor in understanding the pathology of lung adenocarcinoma and warrants further investigation into its potential as a new prognostic factor and therapeutic target.

## Data Availability

De-identified data will be made available upon request for research purposes only and with valid Data Transfer and Use Agreements required for sharing protected human subject data. Please contact the corresponding author regarding data inquiries.
